# Crucial Role for BAFF-BAFF-R Signaling in the Survival and Maintenance of Mature B Cells

**DOI:** 10.1371/journal.pone.0005456

**Published:** 2009-05-06

**Authors:** Melanie Rauch, Roxane Tussiwand, Nabil Bosco, Antonius G. Rolink

**Affiliations:** Developmental and Molecular Immunology, Department of Biomedicine (DBM), University of Basel, Basel, Switzerland; University of Miami, United States of America

## Abstract

Defects in the expression of either BAFF (B cell activating factor) or BAFF-R impairs B cell development beyond the immature, transitional type-1 stage and thus, prevents the formation of follicular and marginal zone B cells, whereas B-1 B cells remain unaffected. The expression of BAFF-R on all mature B cells might suggest a role for BAFF-R signaling also for their *in vivo* maintenance. Here, we show that, 14 days following a single injection of an anti-BAFF-R mAb that prevents BAFF binding, both follicular and marginal zone B cell numbers are drastically reduced, whereas B-1 cells are not affected. Injection of control, isotype-matched but non-blocking anti-BAFF-R mAbs does not result in B cell depletion. We also show that this depletion is neither due to antibody-dependent cellular cytotoxicity nor to complement-mediated lysis. Moreover, prevention of BAFF binding leads to a decrease in the size of the B cell follicles, an impairment of a T cell dependent humoral immune response and a reduction in the formation of memory B cells. Collectively, these results establish a central role for BAFF-BAFF-R signaling in the *in vivo* survival and maintenance of both follicular and marginal zone B cell pools.

## Introduction

The pool of peripheral B cells is continuously replenished by newly-formed immature B cells generated in the bone marrow. In the adult mouse, about 2×10^7^ B cells are produced per day [1], [2]. Following several steps of antigen-independent differentiation and depending upon successful rearrangement of the corresponding genes and expression of the B cell receptor (BCR) protein on their surface, only about 20% of the newly-generated bone marrow B cells migrate to the spleen as immature B cells [3]–[7]. These cells are characterized by a short half-life of about 2–4 days and upon further differentiation steps develop into mature, naïve B cells. It has been shown that upon engagement of their BCR, immature B cells undergo apoptosis whereas mature B cells, under the same conditions, are induced to proliferate [3]–[7]. As for the early stages in the bone marrow, in the periphery the BCR signal is not the only requirement for the progression of B cells along their developmental pathway. The surrounding stromal micro-environment, the presence of appropriate growth factors, as well as their ability to respond to them, are all crucial players in the final maturation steps of developing B cells.

Surface expression of CD93 is a hallmark for immature B cells and on splenic B cells is a phenotypic characteristic for so called transitional B cells [3], [5]. The latter can be further subdivided according to the expression of CD21, CD23, IgM and IgD. Thus, transitional type 1 (T1) cells are CD21^−^ CD23^−^ IgM^high^ and IgD^low^, T2 are CD21^+^ CD23^+^ IgM^high^ and IgD^high^, and T3 are CD21^+^ CD23^+^ IgM^low^ and IgD^high^ cells [3], [5], [6]. Recently, it has been suggested that T3 cells, rather than representing an intermediate in the formation of mature B cells, might identify an independent pool of anergic B cells [8]. Therefore, only T1 and T2 cells would represent the immediate precursors of Follicular and marginal zone B cells, the two major mature splenic B cell subsets.

BAFF (B cell activating factor), a member of the TNF family (also termed TALL-1, THANK, BlyS and zTNF4) and BAFF receptor (BAFF-R) play a fundamental role during the transition from immature T1 to T2 B cells and therefore for the generation of mature B cells in the spleen. This was clearly demonstrated by an almost complete lack of follicular and marginal zone B cells and by a block at the T1 cell stage in BAFF as well as in BAFF-R deficient mice [9]–[13]. In these mice, the B-1 compartment was not affected, indicating that the development of this subset was independent of BAFF-BAFF-R signaling. On the other hand, transgenic mice over-expressing BAFF display an overall increase in all B cell subsets, suggesting that all mature B cells express BAFF-R on their surface or are able to respond to BAFF [10], [14]–[16].

The binding of BAFF to the BAFF-R leads to the activation of the NF-*κ*B pathway and ultimately to the transcription of the anti-apoptotic factor Bcl-2 [17]–[19]. The finding that Bcl-2 over-expression can, to a large extent, rescue the mature B cell compartment in BAFF signaling deficient mice, indicates that Bcl-2 expression induced by BAFF is crucial for the survival of B cells during the transition from immature to mature stages [18].

Since BAFF-R is expressed on all mature peripheral B cells and its signaling promotes *in vitro* survival of immature as well as mature B-2 cells, we hypothesised that BAFF-BAFF-R signaling was also playing a central role in the *in vivo* maintenance of the peripheral mature B cell pool. However, the potential survival role of BAFF in the mature B cell pool is masked in both BAFF and BAFF-R-deficient animals due to the associated developmental block at the T1 stage. Therefore, to address this question, we generated a collection of anti BAFF-R mAbs some of which blocked and others failed to block BAFF binding.

Administration of these blocking antibodies to wild-type mice resulted in an almost complete depletion of follicular B cells and a reduction of about 50% in the MZB cell compartment. Non-blocking antibodies had no, or only minor effects on the mature B cell pool. Moreover, by using FcRγ-deficient or Bcl-2-transgenic mice, we could show that this depletion was Fc-Receptor (FcR) and complement independent. Taken together, beyond its essential role in allowing the developmental progression from immature T1 cells into T2–T3 and mature B cells, we formally demonstrate the essential role of the BAFF-BAFF-R signaling in the long-term survival and homeostasis of mature B-2 and marginal zone B cells.

## Results

### Characterization of anti-BAFF-R monoclonal antibodies

**Figure 1:**
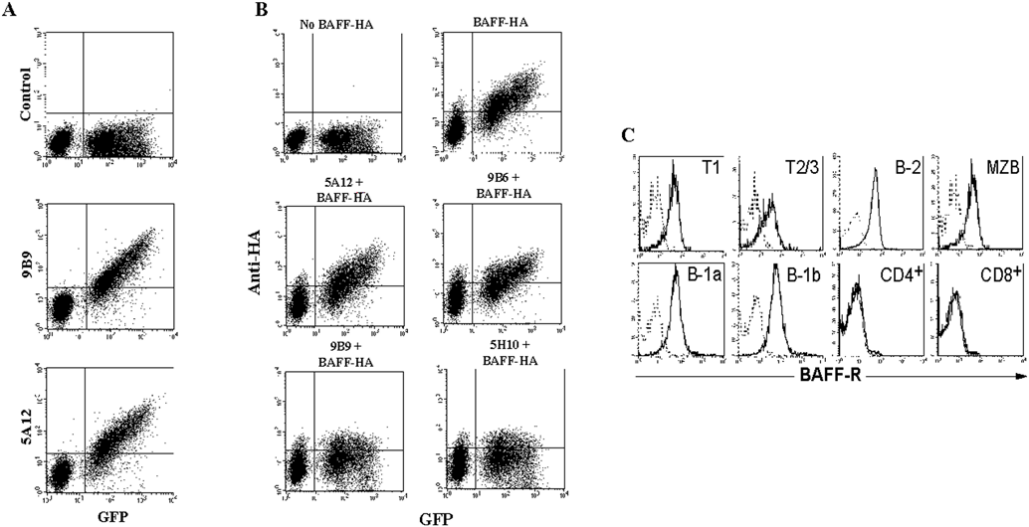
Binding of anti-BAFF-R antibodies to BAFF-R expressing Y3 rat myeloma cells. Panel A. FACS analysis of a 1∶1 mixture of BAFF-R-IRES-GFP transfected and un-transfected Y3 myeloma cells. Upper plot: irrelevant isotype control antibody. Lower plots: the anti-BAFF-R antibodies 9B9 and 5A12 stained the BAFF-R expressing BAFF-R-IRES-GFP transfected Y3 myeloma cells but not the un-transfected GFP negative cells. Panel B. The same mixture of BAFF-R-IRES-GFP transfected and un-transfected Y3 myeloma cells was pre-incubated with or without the four different anti BAFF-R antibodies as indicated: 5A12, 9B6, 9B9 and 5H10 followed by HA-tagged BAFF, which was revealed by a PE labeled anti-HA antibody (upper plot right). 9B9 and 5H10 (lower plots), but not 5A12 and 9B6 (middle plots) were preventing the binding of HA-tagged BAFF to BAFF-R, revealing blocking capacity. Panel C. FACS analysis on the different B and T cell subsets for BAFF-R surface expression, as indicated for each plot. Dotted histograms represent isotype control stainings.

A mixture of un-transfected and mouse BAFF-R-expressing Y3 rat myeloma cells was used to screen supernatants of individual hybridomas generated as described in Materials and Methods. As shown in figure 1A, BAFF-R expressing Y3 (GFP+) cells but not control un-transfected cells (GFP-) stained with two of the generated anti-BAFF-R antibodies, 9B9 and 5A12. After sub-cloning and re-testing, the supernatants of positively-identified clones were used to stain the BAFF-R-expressing Abelson transformed pre-B cell line 40-E1. In total, eleven hybridomas producing anti-BAFF-R mAbs were obtained and of these, five were of rat IgG2a and 6 of IgG2b isotype. To evaluate their blocking capacity, again a mixture of Y3 (GFP−) and BAFF-R expressing Y3 cells (GFP+) was pre-incubated with these mAbs and subsequently with a saturating concentration of HA-tagged BAFF. These experiments were performed using human BAFF, but similar results were obtained using mouse BAFF. FACS analysis with an anti-HA mAb then allowed us to determine which of the generated anti-BAFF-R mAbs blocked BAFF binding. As depicted in figure 1B, pre-incubation of BAFF-R-expressing Y3 cells with mAbs 5A12 and 9B6 did not affect BAFF binding whereas, mAbs 9B9 and 5H10 could block BAFF binding. Of the eleven anti-BAFF-R mAbs generated, five were able to block BAFF binding.

All BAFF-blocking and non-blocking mAbs were used to reveal the expression of BAFF-R on ex vivo isolated spleen, lymph-node, bone marrow, peripheral blood and peritoneal cells. All mature B cells, irrespective of their localization within the lymphatic compartments, namely B-2, MZB, B-1a and B-1b B cells as well as the three immature transitional splenic B cell subsets (T1, T2 and T3) expressed similar levels of BAFF-R (figure 1C). Bone marrow precursor B cells and haematopoietic cells of other lineages did not express detectable surface BAFF-R (figure 1C and data not shown).

### *In vivo* depletion of circulating mature B cells with anti-BAFF-R mAbs that block BAFF binding

**Figure 2:**
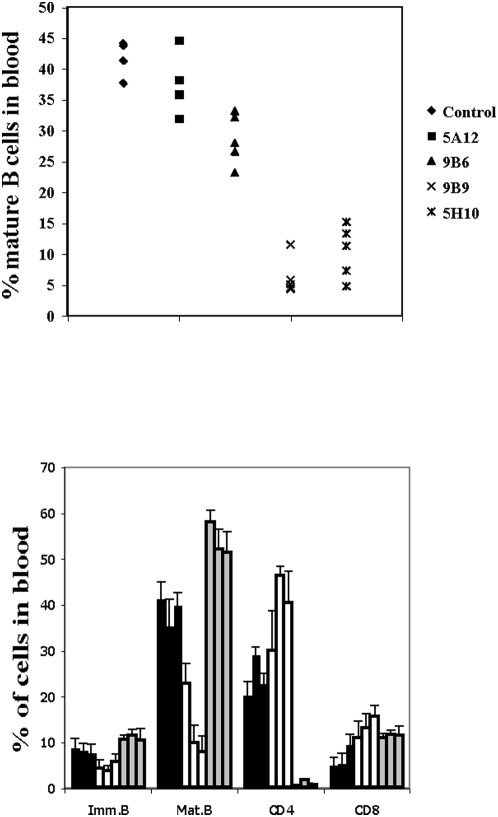
Circulating mature B cells and kinetic analysis of peripheral blood B and T cell depletion. Upper graph: C57BL/6 mice were injected i.v. at day 0 with 0.5 mg of a given anti-BAFF-R antibody, as indicated. At day 14 the percentage of CD93^−^CD19^+^ mature B cells was determined by FACS analysis on the peripheral blood mononucleated cells. Each symbol represents an individual mouse. Statistical analysis revealed a significant difference for control versus 9B6, 9B9 and 5H10 (p<0.05). Lower graph: C57BL/6 mice were injected with either 0.5 mg of the anti-BAFF-R mAb 9B9 (white bars), or 0.5 mg of the anti-CD4 mAb GK1.5 (grey bars). Black bars represent PBS injected controls. The mAbs were injected at day 0 and the percentages of immature B cells (CD93^+^CD19^+^), mature B cells (CD93^−^CD19^+^), CD4 and CD8 T cells were determined by FACS at days 4, 7 and 10. Each column represents a time point: day 4 left column, day 7 middle and day 10 right column. Statistical analysis revealed a significant difference between PBS treated mice as compared to anti-BAFF-R mAb at each time point analyzed for mature B cells but not for immature B cells.

Since BAFF was shown to be a potent survival factor for mature and immature B cells *in vitro*, we reasoned that the *in vivo* use of blocking anti-BAFF-R mAbs would affect the B cell pool. Therefore wild-type C57BL/6 mice were treated with two BAFF-blocking and two BAFF non-blocking anti-BAFF-R mAbs; all four of the same isotype. At day 14 after treatment, the percentage of mature circulating peripheral blood B cells, characterized as CD19^+^ CD93^−^ cells, was determined by flow cytometry. Mature B cells in the control group of PBS treated mice represent about 40% of the circulating leukocytes. Similar percentages were obtained with the non-blocking 5A12 mAb (figure 2A). For 9B6, also a non-blocking mAb, mature B cells ranged from 25–35%, whereas treatment with either 9B9 or 5H10 mAb, both of which blocked BAFF binding, resulted in a dramatic decrease of up to 80–90% of circulating B cells (figure 2A). Therefore, whereas non-blocking mAbs had only a minimal or no effect, the use of mAbs that prevented BAFF binding drastically reduced circulating peripheral B cell numbers. Taken together, these results suggest that mature, circulating B cells require BAFF for their survival.

In order to determine the kinetics of mature B cells depletion, C57BL/6 mice were injected with the BAFF-blocking mAb 9B9 and at days 4, 7 and 10 blood lymphocyte subpopulations were analyzed. Mice injected with either PBS or the *in vivo*-depleting anti-CD4 mAb (GK1.5, rat IgG2b) were used as negative and positive controls, respectively. Results are summarized in figure 2B. Already by day 4 after treatment with GK1.5, almost all CD4^+^ T cells had disappeared and as a consequence, the other lymphocyte sub-populations had proportionately increased. Treatment with 9B9 resulted in a 40% depletion of mature B cells at day 4, which increased to 70% by day 7 and reached its maximal level of 80% by day 10. In accordance with the *in vivo* expected half-life of IgG antibodies, B cell numbers started to recover by day 25–30 after antibody treatment (data not shown).

### The BAFF-BAFF-R interaction is essential for the maintenance of circulating mature B cells

**Figure 3:**
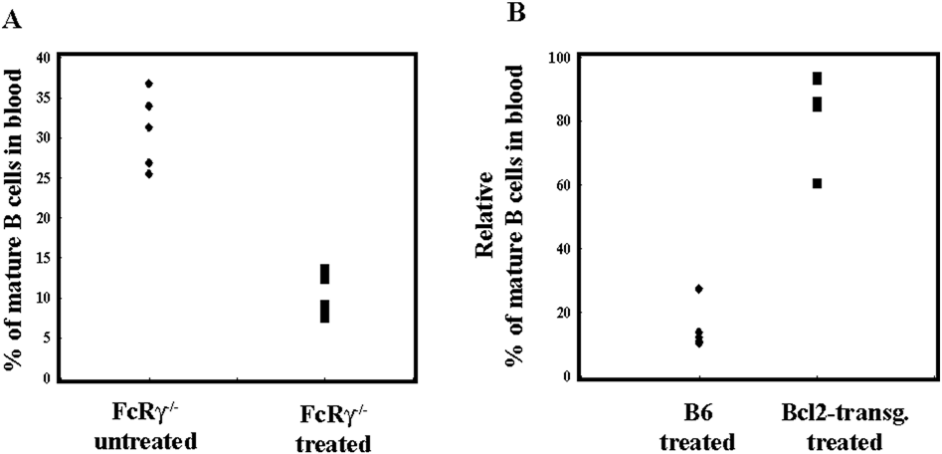
Circulating mature B cells in FcRγ deficient and Bcl2-transgenic mice following anti BAFF-R 9B9 injection. Panel A. FcRγ^−/−^ mice were injected at day 0 and the percentage of CD93^−^CD19^+^ mature B cells in the blood was determined at day 14 by FACS analysis. Non-injected FcRγ^−/−^ mice were used as controls. The difference between FcRγ^−/−^ treated versus untreated mice was statistically significant (p<0.05). Panel B. The percentage of mature B cells in the blood of C57BL/6 and Bcl2 transgenic mice at day 14 following the injection of 9B9 mAb. The difference between C57BL/6 and Bcl2 transgenic mice following 9B9 treatment was statistically significant (p<0.01).

The finding that mAbs that prevented BAFF binding caused a pronounced depletion of circulating B cells whereas, isotype-matched non-blocking ones had only a minor effect, strongly suggested that this ablation was neither due to antibody-dependent cellular cytotoxicity (ADCC) nor to complement-mediated depletion. In order to test this hypothesis, FcR common γ chain-deficient mice were injected with the BAFF-blocking mAb 9B9 or the non blocking mAb 5A12. At day 14 after injection, the percentage of circulating mature B cells (CD19^+^CD93^−^), was determined in the blood and compared to untreated mice. In untreated or 5A12-treated mice, a similar percentage of circulating mature B cells was detected, namely 35% and 25–38%, respectively. In contrast, there were only 7–12% circulating B cells in FcR γ-deficient mice treated with the BAFF-blocking mAb 9B9 (Figure 3A). This result shows that the depletion of circulating mature B cells with BAFF-blocking mAb is FcR independent.

It has been shown that BAFF-induced B cell survival is achieved through an NF-κB mediated increase in anti-apoptotic molecules, including members of the Bcl-2 family. Based on this finding, we wondered whether B cell depletion with BAFF-R blocking mAbs would still be seen in transgenic mice over-expressing Bcl-2. Therefore, C57BL/6 and Bcl-2 transgenic mice were injected with the 9B9 mAb and analyzed after 14 days. As shown in figure 3B the mature B cell pool in the blood of C57BL/6 mice treated with 9B9 was reduced by 80–90%. In marked contrast, only a 5–10% reduction was observed in 4 out of 5 Bcl-2 transgenic animals (figure 2B). Transgenic over-expression of the anti-apoptotic Bcl-2 gene was therefore able to overcome to a large extent, the B cell depleting effect of blocking anti-BAFF-R mAbs. Collectively, these results rule out a role for either ADCC or complement-mediated lysis in the observed depletion of re-circulating B cells and strongly suggest that interactions between BAFF and BAFF-R are crucial for the survival and maintenance of the mature B cell pool.

### BAFF is a survival factor for B-2 and marginal zone B cells *in vivo*

**Figure 4:**
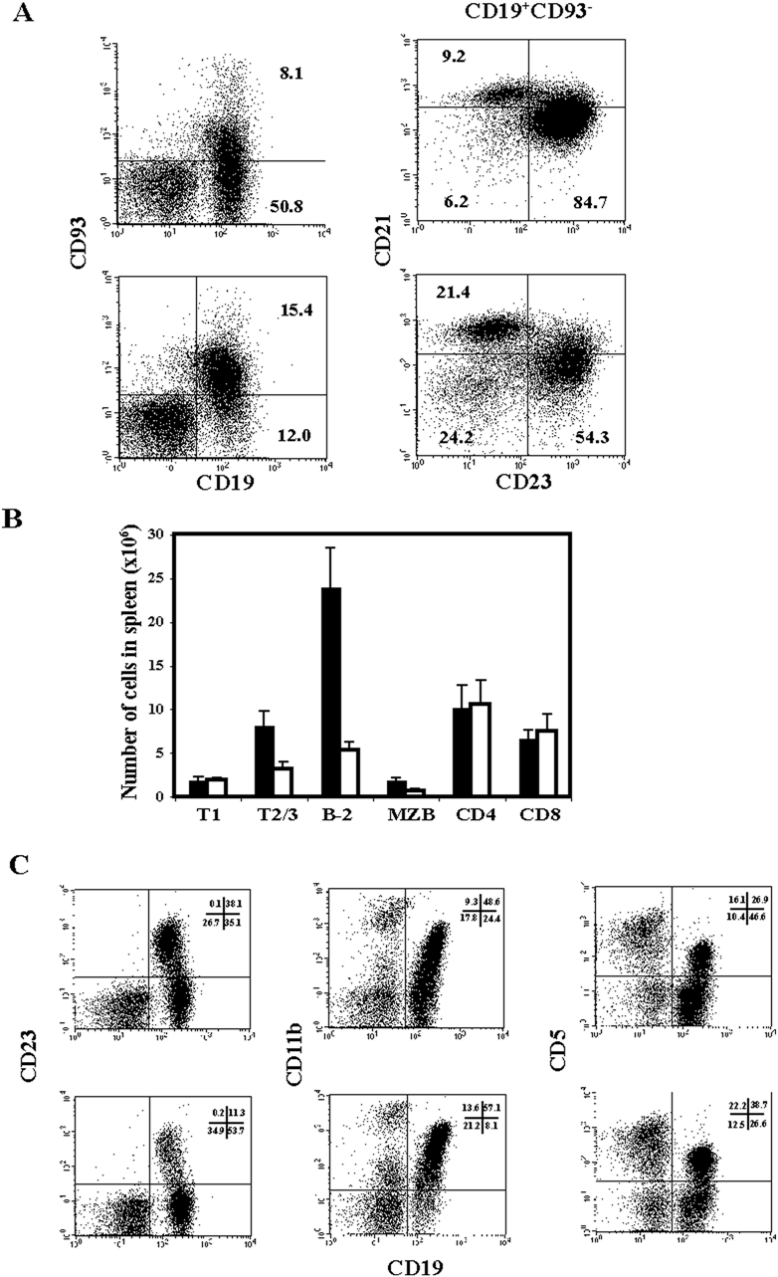
Depletion of the B and T cell subsets following anti BAFF-R 9B9 antibody injection. Panel A. Representative FACS plot of the immature (CD93^+^CD19^+^) and mature (CD93^−^CD19^+^) B cell compartments in the spleen of C57BL/6 mice; upper plots untreated control, lower plots day 14 after 0.5mg of anti-BAFF-R 9B9 injection. On the left side total splenocytes are depicted. On the right side, gated on mature B cells (CD93^−^CD19^+^) follicular (CD21^+^CD23^+^) and MZB (CD21^high^CD23^low^) B cells are shown. Panel B. Absolute numbers of splenic T1 and T2/3 immature B cells, B-2 and MZ B cells, CD4 and CD8 T cells in controls (black bars) and 9B9 injected C57BL/6 mice at day 14 after injection (white bars). 5 mice were analyzed for each group. A significant difference could be observed for T2/T3, B-2 and MZB cell numbers in control as compared to 9B9 injected mice. Panel C. Representative FACS plot analysis indicating the percentages of CD19^+^CD23^+^ B-2, CD19^+^CD11b^+^ B-1b and CD19^+^CD5^+^ B-1a B cells in the peritoneal cavity of control (upper dot plots) and C57BL/6 mice injected with 9B9 mAb at day 14 after injection (lower dot plots).

Mature, peripheral B cells in the mouse can be subdivided into B-2, also called follicular B (Fol B), MZB and B-1 B cells. In order to determine the effect of a blocking anti-BAFF-R mAb on the different B cell subsets, we injected C57BL/6 mice with the mAb 9B9 and analyzed the bone marrow, spleen, lymph nodes and peritoneal cavity lymphocytes at days 14 to 21. In the spleen, the immature transitional B cell subpopulations were only slightly affected with no reduction of T1 and a two fold reduction in T2/T3 subsets (figure 4A and B). Mirroring the depletion of circulating B cells, injections with the mAb 9B9 resulted in a 4–5 fold reduction of CD93^−^CD19^+^ mature splenic B cells. The highest reduction, 80–90%, was observed among follicular (CD21^+^CD23^+^) B cells, whereas MZB (CD21^high^CD23^low^) cells were only decreased by 50%. The number of CD21^−^CD23^−^ mature splenic B cells, which to a large extent comprises B-1 B cells, was not affected at all. CD4 and CD8 T cells also remained unchanged (figure 4B).

Mature B cells in the bone marrow or lymph nodes, both of which consist almost entirely of B-2 B cells, were also reduced by 80–90% upon 9B9 treatment (data not shown). Bone marrow B cell progenitors were not affected by the treatment (data not shown).

Mature B cell subsets in the peritoneal cavity can be subdivided into B-2 (CD19^+^CD23^+^), B-1a (CD19^+^CD23^−^CD11b^+^CD5^+^) and B-1b (CD19^+^CD23^−^CD11b^+^CD5^−^) B cells (figure 4C upper plots). All these subsets express similar levels of BAFF-R (figure 1C). However, upon 9B9 treatment, whereas 70% of the B-2 B cells were depleted, both B-1a and B-1b cell subpopulations remained unaffected (figure 4C).

**Figure 5:**
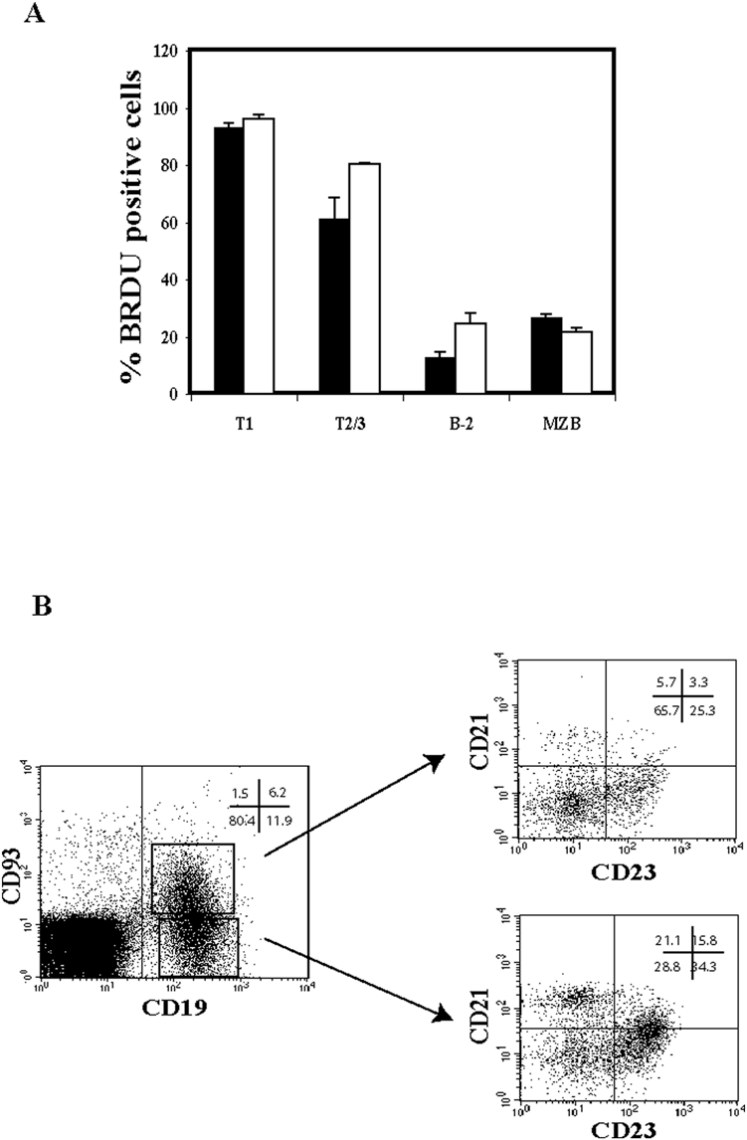
Turnover of splenic B cells following injection with anti-BAFF-R 9B9. Panel A. Turnover of splenic B cell populations in control (black bars) and 9B9 injected C57BL/6 mice (white bars). C57BL/6 mice were injected with 1 mg of BrdU and BrdU was added to the drinking water. 10 days after splenic T1, T2/3, B-2 and MZ B cells were stained, sorted and the percentage of BrdU positive cells was determined by FACS analysis. Mean values with standard deviation are shown. 4 mice were analyzed for each group. Differences were statistically not significant. Panel B. Representative FACS plot analysis of the immature (CD93^+^CD19^+^) and mature (CD93^−^CD19^+^) B cell compartments in the spleen of C57BL/6 mice treated over a 5 months period with anti-BAFF-R 9B9 mAb. Depicted on the right side, CD21 and CD23 staining gated on immature B cells (upper plot) and on mature B cells (lower) plot. Indicated are the percentages of the cells represented in each quadrant.

Taken together, these findings show that maintenance of the vast majority of B-2 and about half of the marginal zone B cells is highly dependent upon the interaction between BAFF and BAFF-R, whereas that of B-1 B cells is largely BAFF-R independent. The fact that some B-2 and about half MZB cells remained following antibody treatment might suggest either that some of these B cells do not require BAFF-BAFF-R interaction for their survival or that B-2 and MZB cells are constantly re-generated having a high turnover rate but are still dependent upon BAFF for their survival. In order to discriminate between these alternatives, we determined the turnover rate of splenic B cell subpopulations in control and 9B9 treated mice by BrdU labeling. In accordance to their high turn-over rate, after 10 days of continuous BrdU labeling, the vast majority of T1 and T2/T3 in control and 9B9 treated mice were found to be BrdU positive (figure 5A), whereas only about 10% of control and 18% of B-2 cells from 9B9 treated mice were BrdU positive (figure 5A). Taken together, the turnover rate of the remaining B-2 cells in 9B9 treated mice was similar to that of B-2 cells in control mice and is indicative of a BAFF-BAFF-R-independent mechanism for the maintenance of this small number of B-2 cells in treated mice. After 10 days of BrdU labeling, about 25% of the MZB cells from the control and 20% from the 9B9 treated mice, were positive (figure 5A).

In order to test whether prolonged treatment would improve the B cell depletion, mice were injected over a 5 months time. FACS analysis revealed that such a prolonged treatment did not alter the outcome of the B cell depletion. Meaning that, B-1 B cell compartment was not affected (data not shown), MZB cells were reduced by half and 10–20% of the B-2 B cell compartment was still present (figure 5B). Thus, the vast majority of B-2 cells are highly dependent for their survival on BAFF-BAFF-R signaling, and only about half of the MZB cells seem to be BAFF-BAFF-R dependent.

### Disturbed splenic architecture and partly impaired humoral immune response in 9B9 treated mice

**Figure 6:**
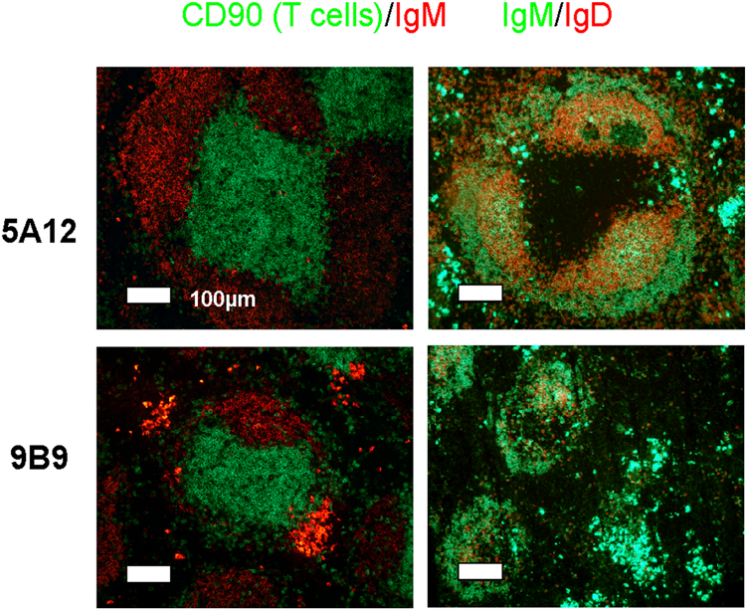
Immunohistochemistry of spleen sections. Spleen histology of C57BL/6 mice 14 days after injection of either the non-blocking 5A12 or blocking 9B9 anti-BAFF-R mAbs, as indicated. Cryosections were stained with anti-IgM (red) and CD90 (T cells) (green), left panels, and with anti-IgM (green) and anti-IgD (red), right panels, as indicated above. Magnification 240×.

In the spleen of normal mice, IgM^low^ IgD^high^ follicular B (B-2) cells are clustered in B cell follicles and are surrounded by a rim of IgM^high^ IgD^low^ marginal zone B cells (figure 6). Plasma cells, characterized by strong cytoplasmic IgM expression, are localized outside the follicles within the red pulp. In mice treated with the BAFF-R blocking mAb 9B9 splenic B cell follicles were considerably smaller (figure 6) as a consequence of the drastic reduction of the follicular B cells. T cell areas did not show a significant reduction in size, confirming the results obtained from the FACS analysis (figure 6). As in controls, plasma cells in anti-BAFF-R treated mice were localized in the red pulp and were identical in numbers, as determined by ELISpot assay (data not shown). Collectively, these results show that treatment with a blocking anti-BAFF-R mAb perturbs the splenic follicular organization by severely depleting mature B cell numbers and thereby reducing follicular size.

**Figure 7:**
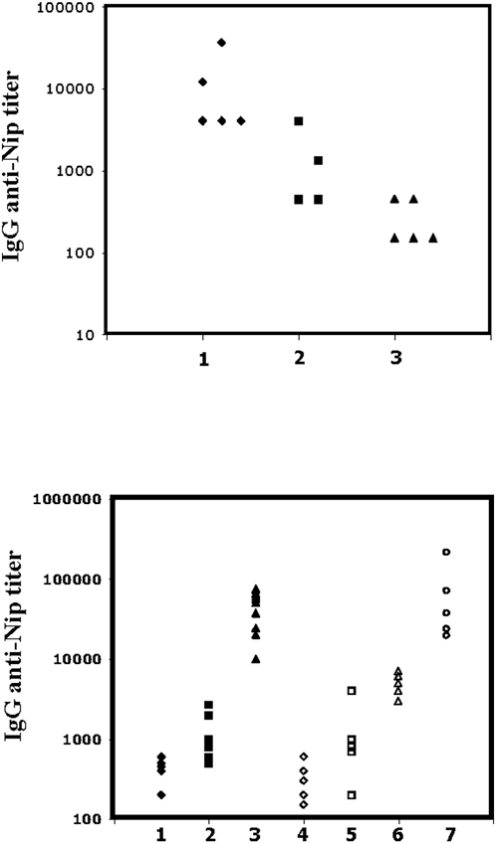
Humoral immune response. Panel A. 12 days after T dependent immunization serum levels of anti-NIP IgG were measured by ELISA in groups of 5 controls and 5 C57BL/6 mice injected either with anti-BAFF-R mAb 9B9 or anti-CD4 mAb GK1.5, as indicated. Immunization with NIP-ovalbumin was performed 10 days after injection of the 9B9 or the GK1.5 mAbs. The titer is defined as the serum dilution that gives OD values twice the background and is depicted on a logarithmic scale. Each symbol represents a mouse. A significant difference could be observed in the response of 9B9 as well as GK1.5 mAbs treated mice as compared to untreated. Panel B. Sera of mice subjected to different treatments was collected at different time points as indicated for each group. Mice received repeated injections of the mAb every third week, over the indicated period of time. Levels of anti-NIP IgG were measured. Each symbol represents a mouse. The titer is defined as the serum dilution that gives OD values twice the background and is depicted on a logarithmic scale. Groups 1 and 4: serum collected at day 1. Mice were treated with 5A12 mAb (non-blocking) and 9B9 mAb (blocking), respectively from day 1 to day 14. Mice were not immunized. Groups 2 and 5: serum was collected at day 74. Mice were treated with 5A12 mAb (non-blocking) 9B9 mAb (blocking), respectively from day 1 to day 74. Mice were only boosted at day 60. Groups 3 and 6 Serum was collected at day 74. Mice were treated with 5A12 mAb (non-blocking) and 9B9 mAb (blocking), respectively from day 1 to day 74. Mice were primed with NIP-ovalbumin at day 14 and boosted at day 60. Group 7. Serum was collected at day 94. Mice were treated with 9B9 mAb (blocking) from day 60 to day 74. Mice were primed with NIP-ovalbumin at day 14 and boosted at day 80. Statistical analysis revealed a significant difference for group 3 versus group 6, but not for group 3 versus 7.

In order to test whether the B cell depletion and alteration of the splenic architecture, induced by the treatment, would influence humoral immune responses, mice were immunized with NIP-ficoll or NIP-ovalbumin 10 days after treatment with 9B9 mAb. Serum IgM and IgG anti-NIP titers were determined at day 12 after antigen administration. Depletion, following anti-BAFF-R treatment was not affecting IgM titers upon challenge with T cell independent NIP-ficoll antigen (data not shown), whereas, a 5 to 10 fold reduction in the titers was observed in 9B9 treated compared to control animals, following a T dependent NIP-ovalbumin (NIP-OVA) immunization (figure 7A). However, 9B9 injected mice were still able to mount a significantly higher immune response (3–5 fold), compared to mice depleted of CD4 T cells by GK1.5 mAb injections (figure 7A). Consistent with this finding, the presence of small germinal centers could be detected in 9B9 treated mice after immunization by histological analysis (data not shown). Thus, mice treated with an anti-BAFF-R mAb which prevents BAFF binding showed an impaired but not a completely abrogated ability to mount a primary antigen specific IgG response. To evaluate the impact of B cell depletion on the formation of memory B cells mice were injected with the 9B9 blocking mAb or the 5A12 control non-blocking mAb 14 days before being immunized with NIP-OVA. The Ab treatment was continued over a two months period and at day 60 after priming mice were boosted, and the recall IgG anti-NIP response was determined at day 74. As shown in figure 7B (3 versus 6) the IgG anti-NIP titer of the memory response was 5–10 fold lower in the 9B9 treated group (figure 7B group 6) as compared to the 5A12 treated group (figure 7B group 3). Thus, treatment with an anti-BAFF-R mAb that prevents BAFF binding impaired the formation of memory B cells. Nevertheless the induction of memory formation was not completely abrogated by 9B9 treatment, since the antigen specific IgG response was still higher as compared to not immunized (figure 7B group 1), not immunized 9B9 treated (figure 7B group 4) mice as well as mice which received only the booster immunization, irrespective of the 9B9 treatment (figure 7B group 2 and 7B group 5, respectively).

To address the role of BAFF-BAFF-R signaling on the maintenance of memory B cells, NIP-OVA primed mice were injected with 9B9 mAb at day 60 and 74 following immunization. At day 80 after priming mice were boosted and the IgG anti-NIP titer was determined at day 94. As shown in figure 7B group 7, the mice mounted an IgG anti-NIP response that was not significantly different from control treated mice (figure7B group 3). Collectively, these results suggest that formation of memory B cells requires BAFF-BAFF-R signaling, while the maintenance and survival of memory B cells is not affected by 9B9 treatment and therefore BAFF independent.

## Discussion

The role of BAFF in the development of mouse B cells was most clearly demonstrated by the generation of two different BAFF deficient strains and by the characterization of BAFF-R deficient mice [9]–[13]. These mutant mice displayed a severe block in B cell development at the differentiation from, so called, T1 to T2 B cells in the spleen, whereas the development of B-1 B cells appeared to be unaffected. As a consequence of the profound decrease in T2 B cell numbers, their downstream mature B cell progeny, namely follicular and marginal zone B cells, were drastically depleted. Whether BAFF was necessary either for the survival of T2 cells or for promoting their differentiation and maturation, however, remained an open question. Furthermore, the role of BAFF in the survival of mature B cell *in vivo* could not be addressed using these mice, since their precursors depended on BAFF for their generation and development.

By the generation and administration of anti-BAFF-R monoclonal antibodies capable of preventing BAFF binding, we show in this report, that the *in vivo* survival of almost all follicular and half of the marginal zone B cells is dependent upon BAFF-BAFF-R signaling. Injection of mice with a blocking anti-BAFF-R antibody induced a profound depletion of the mature B cell compartment, whereas a non-blocking antibody had only a minimal or no effect. The possible scenarios which could explain this phenomenon include: an antibody-dependent cellular cytotoxicity (ADCC), a complement-mediated lysis, an impairment of the survival through prevention of BAFF binding or a limited generation of newly formed mature B cells as a consequence of the depletion of transitional B cells. The fact that an isotype-matched non-blocking anti-BAFF-R monoclonal antibody is not affecting the peripheral mature B cell pool and that in addition, the detectable presence of the non blocking anti-BAFF-R antibody on the surface of non-depleted B cells (data not shown), taken together would indicate that an ADCC mechanism is unlikely. Moreover, that B cell depletion is still occurring in FcR-γ-chain [20] deficient mice confirms that ADCC is most likely not involved. In comparison to the rapid ADCC dependent anti-CD4-mediated depletion of T cells, the relatively slow kinetic of B cell deletion observed after the administration of blocking anti-BAFF-R antibody suggested a major role for BAFF-R signaling on mature B cell survival. Moreover, treatment of Bcl-2 transgenic mice [21], where B cells were only slightly reduced, further corroborates the hypothesis of a BAFF mediated survival on the one hand and makes complement mediated cell lysis improbable on the other hand.

Yet an alternative explanation for the observed depletion of mature B cells upon treatment would be that the numbers of differentiating T2/T3 immature B cells into mature cells decrease with time. However based on BrdU *in vivo* labeling studies performed by us and others this seems to be an unlikely scenario [3], [7]. These studies showed that in an adult mouse about 1% of the mature B-2 cells are replaced per day. Upon treatment we see already an 80–90% reduction of mature B cells at day 10–14, whereas only a 10–14% reduction could be explained by the differentiation block of T2/T3 into mature B-2 cells. Therefore collectively our findings demonstrate that the vast majority of B-2 cells and about half of MZB cells require BAFF for their survival.

Different experimental approaches, performed by other groups, were also suggestive of a survival role of BAFF-BAFF-R signaling in mature B cells. Treatment of mice with a TACI-Fc fusion protein was shown to lead to a reduction of B cells [22]–[24]. However, in these studies the extent of B-2 and MZB cells depletion was not analyzed in detail [22], [23]. Moreover, given that TACI can interact with both BAFF and APRIL [24], the potential role for APRIL in this depletion process could not be excluded. Several groups showed that treatment of mice with a BAFF-R-Fc fusion protein also resulted in a depletion of B cells [22], [25]. Since BAFF is the only known ligand for BAFF-R these studies strongly suggest that peripheral B cells require BAFF for their survival. In one of these studies a more detailed analysis of the extent of depletion of the various mature B cell subpopulations is reported [25]. This analysis revealed that after such a treatment the B-2 and MZB compartments were largely reduced whereas the B-1 cell numbers were practically not affected. In other words, treatment of mice with a BAFF-R-Fc fusion protein results in a very similar B cell depletion as we observed here upon treatment with the blocking anti-BAFF-R mAbs. Thus, our results confirm and extent previously reported findings on the role of BAFF-BAFF-R signaling in the survival and maintenance of the mature B cell compartments.

B-1 B cells express relatively high amounts of BAFF-R on their surface. However, as shown in BAFF as well as BAFF-R deficient mice their generation and maintenance is not affected [9]–[13]. Also the here described short-term and long-term (5 months) treatment with anti-BAFF-R mAbs that block BAFF binding did not affect the B-1 B cell compartment. Moreover, we could rule out an inability of the injected mAbs to enter the peritoneal cavity since FACS analysis with an anti-rat IgG revealed the presence of the anti-BAFF-R mAb on the surface of B-1 B cells (data not shown). Thus the role of the BAFF-R in B-1 B cell biology still needs to be elucidated. However the findings that the B-1 B cell compartment is largely expanded in BAFF transgenic mice [10], [14]–[16] might suggest that BAFF can act as a B-1 B cell growth factor.

Following anti-BAFF-R treatment, we observed that the B-2 B cell compartment was the most affected B cell subset, indicating that the majority of B-2 cells rely on BAFF signaling for their survival. Marginal zone B cells were reduced only by half upon treatment, which compared to their almost complete absence observed in BAFF and BAFF-R deficient mice, is indicative for a crucial role of BAFF signaling during marginal zone B cell development or survival of their progeny, but dispensable for their survival subsequent to maturation. In BAFF-R deficient mice, over-expression of Bcl-2 could not overcome the marginal zone B cell defect [18], arguing for an instructive role of BAFF for their development, which still needs to be elucidated.

By BrdU labeling experiments and FACS analysis we show that the survival of a small subset of B-2 B cells seemed to be BAFF-independent and not reflecting newly formed mature B cells. A similar result was shown in BAFF as well as BAFF-R deficient mice, where the mature follicular B cell compartment was drastically reduced but still present in small numbers [26]. The follicular B cells that survived this BAFF-R blockage could not be distinguished according to phenotypic criteria (data not shown). Because B-1 cells were not affected by anti-BAFF-R treatment and considering their predominant origin during fetal/neonatal development, we wondered whether the surviving B-2 cells were also of fetal origin. A hallmark of B cell development during fetal life is the lack of expression of deoxynucleotidyl transferase which prevents non-templated nucleotide additions in the V-D and D-J junction of the BCR heavy chain. No difference could be observed comparing junctional regions of untreated to 9B9 treated B-2 B cells, ruling out this hypothesis (data not shown).

Prolonged (5 months) treatment with the 9B9 mAb did not improve the depletion of these mature B cell subsets. FACS analysis with an anti-rat IgG mAb revealed the presence of 9B9 on the surface of B-2 and MZB cells surviving the treatment (data not shown). Moreover, the surface available BAFF-R seemed to be saturated by the injected 9B9, as anti-BAFF-R mAbs were undetectable by FACS analysis (data not shown). Therefore, the survival of these mature B cell subsets seems to be BAFF–BAFF-R signaling independent.

It has been shown that surface BCR expression is mandatory for mature B cell survival, since conditional ablation of BCR expression subsequent to the establishment of steady-state B cell numbers, resulted in rapid death of most peripheral B cells [27]. The mechanism by which BCR expression influences B cell longevity remains to be clarified.

The survival of mature B cells is dependent on signaling processes that use the NF-κB signal transduction pathway [28], [29]. Two pathways leading to NF-κB activation in B cells have been described, namely the classical and the alternative pathways [30]. Several mutations affecting one or both NF-κB signaling cascades were shown to affect the B cell compartment [12], [31]–[37]. Since the two NF-κB pathways employ both shared and distinct components, the role of each activation pathway in B cells remains to be elucidated [18].

Several *in vitro* studies have demonstrated a survival role of BAFF on transitional as well as mature B cells [16], [38]. The mechanism by which this increased survival is achieved seems to be dependent on the NF-κB mediated up-regulation of anti-apoptotic Bcl-2 family proteins and inhibition of the nuclear translocation of the pro-apoptotic protein kinase Cδ [39], [40]. Collectively, BCR as well as BAFF-R signaling seem to be essential for the maintenance of mature B cells and this is probably achieved by up-regulating anti-apoptotic pathways, where Bcl-2 might be a key player. In fact, transgenic expression of Bcl-2 was able to rescue the survival of B cells upon BCR deletion [27], and we show that it is able to overcome, to a large extent, the ablating effect of anti-BAFF-R blocking antibodies. Mature B cell survival seems to be regulated by the achievement of a certain activation state induced by a combination of basal BCR and BAFF-BAFF-R signaling, with a different contribution of the two pathways which might also be dependent on the B cell subset. Nevertheless, it still remains to be clarified how BCR and BAFF-R signals act on one another and are integrated within the cell to maintain cell survival. The different impact of BCR and BAFF-BAFF-R signaling on the activation state and as a consequence on the survival of mature B cells could explain why some B cells survive anti-BAFF-R treatment as a BAFF-independent subset. In agreement with this hypothesis is the finding that many marginal zone B and B-1 B cells survived treatment with anti-BAFF-R mAb.

It is believed that formation of the different mature B cell compartments is influenced by specific BCR-ligand interactions [41]–[43]. Thus, B cells in transgenic mice expressing recombinant BCRs for self antigens tend to differentiate into B-1 and marginal zone B cells [44]. Both, B-1 and MZB cells are enriched for self reactive clones, whereas follicular B cells generally require higher levels of BCR signaling for their formation. Therefore, if for the survival of mature B cells a certain threshold of activation is required and if this activation is a combination of basal BCR signaling and competition for as well as the availability of BAFF, prevention of BAFF binding would rather favor the survival of those B cells with relatively higher affinity for self antigens, such as B-1 and MZB cells. Nevertheless, we cannot rule out an alternative explanation for the BAFF independent survival of MZB cells, namely that their location next to metallo-phillic macrophages could provide them with a specific environmental niche favoring their survival. Moreover, considering that their development is dependent on Notch signaling [45], [46], different Notch ligands might also be involved in their maintenance.

As a consequence for the observed B cell depletion in anti-BAFF-R treated mice, we show that splenic follicles are greatly reduced in size. Moreover, the primary immune response to T dependent antigen was impaired, with IgG titers reduced by a factor of 5 to 10 following treatment with an anti-BAFF-R blocking antibody, whereas total immunoglobulin levels were not affected. This result is in line with what was shown for BAFF-R deficient mice, where the IgG_1_ response to T-dependent antigen was significantly reduced [12]. Moreover, we showed that in absence of BAFF-R signaling the induction memory B cells is strongly impaired. However, the maintenance of memory B cells seems to be BAFF–BAFF-R signaling independent since 9B9 mAb treatment of mice primed before with a T cell dependent antigen did not impair the recall response. This finding confirms the very recent data published by Benson et al. [22] showing that treatment of antigen primed mice with TACI-Ig or BAFF-R-Ig does not impair their ability to mount an efficient recall response.

These findings enlighten a new role of BAFF-BAFF-R signaling as a crucial factor for the formation of memory or the survival of developing memory B cells, while confirming its dispensable role in the maintenance of memory B cells. Therefore the question whether therapies, based on BAFF as well as BAFF-R neutralization in B cell mediated autoimmune diseases, could be successful remains uncertain. On the other hand, since elevated BAFF serum levels and deregulated BAFF-R signaling were shown to contribute to the pathogenic B survival in oncological as immunological disorders [47]–[52], a potential use of anti-BAFF-R mAb might represent an optimal targeted therapy, which would not compromise the ability of these patients to respond to already encountered antigens.

## Materials and Methods

### Experimental animals

Female C57BL/6 and Lewis rats were purchased from RCC Ltd., (Füllinsdorf, Switzerland). FcRγc^−/−^ mice were obtained from Taconic (Ejby, Denmark). Bcl-2 transgenic mice [21] were obtained from Dr. A. Trump (ISREC, Lausanne, Switzerland) and were bred under pathogen free conditions at the Center for Biomedicine, Basel. All animal experiments were carried out within institutional guidelines with the permission of the national and local authorities (the permission number for the principal investigator are 1886, 1887 and 1888).

### Antibodies and flow cytometric analysis

FITC-, PE-, APC-Cy7-, PE-Cy7- or biotin-conjugated mAbs specific for CD4 (RM4-5), CD5 (53-7.3), CD8α (53-6.7), CD11b (M1/70), CD19 (1D3), CD21 (CR2/CR1; 7G6), CD23 (B3B4) and CD45R (B220; RA3-6B2) were purchased from BD Biosciences (BD Pharmingen™). Biotin-labeled Mouse anti-Rat IgG was purchased from Jackson ImmunoResearch Laboratories, Europe LtD. Antibodies specific for IgD (1.19), IgM (M41), CD90 (Thy-1; T24), CD93 (C1qRp; PB493) and HA-peptide (12CA5) were purified from hybridoma supernatants and labeled with FITC or biotin using standard procedures. Biotin-labeled antibodies were revealed by PE- or PE-Cy7-Streptavidin (BD Biosciences). Staining of cells was performed as described previously [53]. *In vivo* BrdU labeling and subsequent analysis was performed as described [3]. Flow cytometry was performed using a FACS Calibur (BD Biosciences) and data were analyzed using the Cell Quest Pro Software (BD Biosciences).

### Cloning, expression and purification of soluble human BAFF

A soluble form of human BAFF was expressed as a HA-tagged molecule from Drosophila SL-3 cells as previously described [54]–[56], briefly a fragment corresponding to the soluble C-terminal part of the human BAFF was cloned into the ApaI-NotI-opened expression vector pRmHa-3 HA/myc/TM (a kind gift of Dr. K. Karjalainen, NTU School of Biological Sciences, Singapore), yielding the pRmHa-3 HA-hBAFF plasmid.

### Cloning of the mouse BAFF-R gene and its expression in rat and mouse B cell lines

Full-length mouse BAFF-R cDNA (GenBank accession number: NM_028075) was amplified from mouse spleen cDNA using primers mBAFF-R4 (5′-ATT AGA TCT GAA ATG GGC GCC AGG AGA CTC C-3′) and mBAFF-R5r (5′-GAT GAA TTC CTA TTG CTC TGG GCC AGC TG-3′). The PCR fragment was digested with BglII and EcoRI and cloned into the BglII and EcoRI opened pMIG plasmid (Addgene plasmid 9044), allowing the bi-cistronic expression of BAFF-R together with green fluorescent protein (GFP). The retroviral vectors were transfected into the Phoenix retroviral packaging cell line (ATCC® Number: SD 3443); according to the manufacturer's instructions. The rat myeloma cell line Y3 (ATCC® Number: CRL-1631) and the mouse pre B cell line 40E1 (Alt, 1981) were retrovirally transduced by spin-infection using standard procedures. Transduced cells were sorted by FACS ARIA (BD Biosciences) on the basis of high GFP expression.

### Generation of anti-mBAFF-R mAbs

Lewis rats were immunized subcutaneously with 10^7^ Y3-mBAFF-R cells to generate anti-mBAFF-R mAbs as described earlier [3]. Hybridomas were screened for IgG antibodies selectively binding to mBAFF-R. For FACS analysis of BAFF-R expression, purified mAbs were labeled with biotin, Alexa Fluor® 488 or Alexa Fluor® 647 (Invitrogen AG, Basel, Switzerland) according to standard procedures. In order to test whether the generated anti-BAFF-R mAb's were able to block BAFF binding, a mixture of Y3 (non GFP expressing) and Y3-mBAFF-R (GFP expressing) cells was incubated with the various mAbs for 30 minutes on ice. After washing, cells were then incubated 30 minutes with 10 µg/ml of soluble HA-tagged hBAFF. After washing, hBAFF binding was revealed by using the HA-peptide specific mAb 12CA5.

### Treatment of mice with anti-BAFF-R mAbs

Mice were injected intravenously (i.v.) with 0.5 mg mAb in PBS. Lymphocyte subpopulations in the blood, spleen, lymph nodes, bone marrow and peritoneal cavity were analyzed by FACS at various time points after injection. Mice treated with the *in vivo* depleting [57] rat IgG2b anti-CD4 mAb GK1.5 (0,5 mg i.v.) were used as controls.

### Immune responses

Mice were immunized i.p. with 100 µg NIP-OVA (Biosearch Technologies Inc., Novato, CA) in alum (T dependent immune response) or i.v. with 50 µg NIP-FICOLL (Biosearch Technologies Inc., Novato, CA) in PBS (T independent immune response). At day 14 after immunization, the mice were bled and the serum IgG anti-NIP titer was determined by ELISA as previously described [58]. For recall response, 8 weeks after immunization mice were boosted with 10 µg NIP-OVA in PBS and 10 days after serum IgG anti-NIP titer was determined by ELISA.

### Immunohistology

Spleens were snap frozen and embedded into OCT compound (Sakura, Zoetermeer, NL). Cryostat sections of 5 µm were prepared and fixed for 10 minutes in acetone. Sections were stained with anti-IgM^FITC^ (M41) and anti-IgD^Biotin^ (1.19) or anti-Thy1^FITC^ (T24) and anti-IgM^Biotin^ (M41). Biotin-labeled antibodies were visualized using streptavidin Texas red (BD Biosciences). Stained sections were analyzed using an Axioskop Immunofluorescence (Zeis, Feldbach, CH) equipped with a Nikon digital camera.

### Statistical analysis

Differences between groups were evaluated for statistical significance using the two-tailed paired student's t test, assuming equal variances.
